# Fauna Associated with American Alligator (*Alligator mississippiensis*) Nests in Coastal South Carolina, USA

**DOI:** 10.3390/ani14040620

**Published:** 2024-02-14

**Authors:** Thomas R. Rainwater, Randeep Singh, Clarissa A. Tuten, Aaron M. Given, Parker W. Gibbons, Bo Song, Steven G. Platt, Philip M. Wilkinson, Catherine M. Bodinof Jachowski

**Affiliations:** 1 Tom Yawkey Wildlife Center, 1 Yawkey Way S., Georgetown, SC 29440, USA; usalligator55@gmail.com (R.S.); philmwilk@gmail.com (P.M.W.); 2 Belle W. Baruch Institute of Coastal Ecology and Forest Science, Clemson University, Georgetown, SC 29440, USA; bosong@clemson.edu; 3 Department of Forestry and Environmental Conservation, Clemson University, 261 Lehotsky Hall, Clemson, SC 29634, USA; cjachow@clemson.edu; 4 Department of Biology, Coastal Carolina University, Conway, SC 29528, USA; clarissa_tuten@yahoo.com; 5 Town of Kiawah Island, Kiawah Island, SC 29455, USA; agiven@kiawahisland.org; 6 Coastal Carolina Consulting, 1935 Oak Tree Lane, Mt. Pleasant, SC 29464, USA; pwgsnakes@gmail.com; 7 Wildlife Conservation Society-Cambodia Program, #21, Street 21 Sangkat Tonle Bassac, Phnom Penh 12000, Cambodia; sgplatt@gmail.com

**Keywords:** American alligator, *Alligator mississippiensis*, automated game camera, behavior, commensal fauna, ecosystem engineer, nest associate

## Abstract

**Simple Summary:**

The ecological roles and importance of crocodilians (alligators, caiman, crocodiles, gharials) are poorly understood. These large predators are considered to be “ecosystem engineers” because their modification of habitats (e.g., excavation of holes, dens, and tunnels; construction of nests) provides opportunities for vital life activities (e.g., feeding, drinking, breeding, and sheltering) to other wildlife. However, data supporting this contention are scarce for most crocodilian species and, where available, are primarily the result of chance encounters and anecdotal observations; few systematic studies directly focusing on the influence of crocodilian habitat alteration on other wildlife have been conducted. To address this data gap, we monitored American alligator nests with automated game cameras in coastal South Carolina to quantify wildlife visiting nests (faunal associates) and their corresponding behaviors (i.e., how they used nests). From 2016 to 2021, we identified 81 wildlife species at alligator nests, including a wide variety of birds, mammals, reptiles, amphibians, and invertebrates. These animals used alligator nests primarily for feeding/foraging, traveling, and loafing but also basking, burrowing/shelter, breeding, and nesting, and these uses varied by animal group and species. Our results indicate that a diverse assemblage of wildlife uses alligator nest sites in coastal South Carolina for a variety of life activities, and these uses differ among and within animal groups. This study provides a first step for investigations regarding the net impacts of alligator nest-faunal associate interactions and ultimately the greater ecological role of alligators and other crocodilians.

**Abstract:**

Crocodilians are considered to be “ecosystem engineers” because their modification of habitats provides opportunities for feeding, drinking, breeding, and other vital life activities to a wide variety of other animals. One such habitat modification is the construction of nest mounds during the breeding season by most crocodilian species, including American alligators (*Alligator mississippiensis*). While many reports exist describing wildlife associated with alligator nests, no studies have quantified faunal associates and their corresponding behaviors while visiting nests. To address this data gap, we used automated game cameras to monitor wildlife and their behaviors at alligator nests during the egg incubation period (June–September) in coastal South Carolina, USA (2016–2021). We documented a total of 81 species (79 vertebrates and 2 invertebrates) at 78 alligator nests representing six taxonomic groups, including 48 birds (59.2%), 9 mammals (11.1%), 19 reptiles (23.4%), 3 amphibians (3.7%), 1 malacostracan (1.2%), and 1 insect (1.2%). Collectively, faunal associates primarily used alligator nests for feeding/foraging (51.8%), traveling (29.3%), and loafing (19.9%) and to a much lesser extent basking, burrowing/shelter, breeding, and nesting. However, trends in alligator nest use varied among faunal associate groups (birds, mammals, reptiles, amphibians, etc.), subgroups (e.g., passerines, raptors, wading birds, and waterfowl), and species. Several novel behaviors by some nest associates were also noted during the study, including the first observations of Virginia oppossum (*Didelphis virginiana*) opening and predating nests, bobcat (*Lynx rufus*) consuming alligator hatchlings, and Carolina wren (*Thryothorus ludovicianus*) feeding on the contents of a recently predated alligator egg. The results of this study indicate that a diverse assemblage of vertebrates (and some invertebrates) use alligator nest sites in coastal South Carolina for a variety of life activities during the egg incubation period, and the proportion of the behaviors exhibited varies among animal groups and species. This study provides a first step for investigations regarding the net impacts of alligator nest-faunal associate interactions and ultimately the greater ecological role of alligators and other crocodilians.

## 1. Introduction

Organisms that create, modify, or maintain the physical attributes of habitats in ways that influence the distribution, life history, behavior, or abundance of other species are often referred to as ecosystem engineers [1,2]. Though empirical evidence is lacking for many species, crocodilians are generally considered to be ecosystem engineers because their modification of habitats provides opportunities for feeding, drinking, breeding, and other vital life activities to a wide variety of other animals [2,3,4]. Open holes, dens, and tunnels excavated by crocodilians to escape unfavorable environmental conditions and predators serve as reservoirs of fresh water, especially critical during periods of drought or in coastal habitats where other fresh water sources are scarce [2,5,6,7,8,9]. Mound-nesting species build elevated structures (nests) in otherwise flat and often flooded terrain, which can be used as nesting, feeding, and basking platforms by an array of birds, mammals, reptiles, and amphibians [2,10,11,12]. In addition, the construction of a mound nest can result in significant habitat alteration, including a large area (3–7 m in diameter) of disturbance surrounding the mound, disrupted soil substrate, and often a guard hole or den containing water nearby [7,10,13]. Such dramatic changes in habitat over a relatively short period of time (days) may attract or provide opportunities to plants and animals with habitat requirements different from those offered by the surrounding, undisturbed marsh [2,8,9].

American alligators (*Alligator mississippiensis*) are large freshwater crocodilians distributed across the Gulf Coast and lower Atlantic coastal plain of the United States [14]. Nesting occurs during early summer (June–early July) when females construct mound nests, comprising primarily vegetation and soil heaped into a pile and shaped using the legs, tail, and body [13,15,16]. Nests are usually built within a few meters of a lake, pond, canal, or other water source, which later serves as a refugium and nursery habitat for hatchlings [7,13,16,17,18,19]. In coastal habitats where access to fresh water is limited (e.g., barrier or sea islands), nesting often occurs near brackish water or naturally occurring or constructed (“alligator holes”) depressions where rainfall collects [2,7,9,20]. Nesting females deposit a single clutch of up to 75 eggs [21] in the nest mound, and the eggs remain covered inside the nest cavity for the duration of the incubation period [7].

During the incubation period (approximately 66 days), alligator nests are used by a variety of other wildlife (i.e., nest or faunal associates). Nest mounds are often inhabited by insects and small vertebrates [10,19,22] and may be used as nesting sites, feeding platforms, and basking or resting areas by a variety of vertebrates including other alligators, two-toed amphiumas (*Amphiuma means*) [23,24], and multiple species of lizards [25,26,27,28], snakes [10,26,29], turtles [10,25,26,30,31,32,33], birds [10], and mammals [10]. In addition, alligator eggs serve as a food source for multiple vertebrate and invertebrate nest predators including raccoons (*Procyon lotor*) [7,13,15,17,18,19,22,32,34], feral pigs (*Sus scrofa*) [22,35], black bears (*Ursus americanus*) [18,36], river otters (*Lontra canadensis*) [36], Virginia opossums (*Didelphis virginiana*) [15], invasive tegus (*Tupinambis merianae*) [37], and red imported fire ants (*Solenopsis invicta*) [22,38,39]. 

While multiple reports exist describing alligator nest associates, most accounts are the result of chance encounters and observations; indeed, apart from reports by Merchant et al. [10] and Hunt and Ogden [18], no systematic studies of alligator nest associates have been conducted. In addition, to the best of our knowledge, data on fauna associated with alligator nests are only available from the southern portion of the animal’s range (i.e., Texas, Louisiana, Georgia, and Florida); none exist from habitats near the alligator’s northern distributional limit. To address this data gap, we used automated game cameras to monitor wildlife associated with alligator nests in coastal South Carolina from 2016 to 2021. Specifically, the objective of this study was to quantify fauna and their associated behaviors at alligator nests during the egg incubation period to assess how different animal groups use nest mounds and sites.

## 2. Materials and Methods

This study was conducted on the Cat Island and South Island portions (6033 ha) of the Thomas A. Yawkey Wildlife Center (YWC) located on the north-central coast of South Carolina (33° N, 79° W) in Georgetown County [40,41]. YWC consists of tidal wetlands, maritime and pine forests, sand beaches, and tidal managed impounded wetlands (ponds) [40,41]. Most alligator nesting on YWC occurs in or closely adjacent to managed impounded ponds (1012 ha), which are typically maintained at water levels <60 cm deep, vegetated with widgeon grass (*Ruppia maritima*), a submerged aquatic plant and preferred waterfowl food, and interspersed with emergent tall cordgrass (*Spartina cynosuroides*), salt marsh bulrush (*Scirpus robustus*), and smooth cordgrass (*Spartina alterniflora*) [7,40]. Water salinity in impoundments ranges from 0 to 35 ppt, depending on rainfall and water management practices [7,40]. 

From 2016 to 2021, we conducted 2–3 weekly helicopter surveys during the alligator nesting season (early June–early July) to locate alligator nest mounds at YWC. Upon discovery from the air, global positioning system (GPS) coordinates were collected for each nest while hovering over the mound. Each nest was then located on foot within 3 to 72 h and opened to confirm oviposition. Empty nest mounds were re-visited daily until oviposition occurred or until the nest was determined to be false or abandoned. At each nest, we mounted a single automated game camera (primarily Reconyx XR6 Ultrafire, Holmen, WI, USA, but in some cases Moultrie Game Spy 2 Plus, Birmingham, AL, USA) on a steel fence post (2016–2018), wooden stake (2017–2018), or custom-made wooden camera stand (2019–2021), all within 3.96 m (13 ft) of the nest. From 2016 to 2018, we activated motion sensors on cameras to photograph alligator nest associates; however, we discovered that in many cases, these passive infrared sensors failed to detect attending female alligators (and likely other ectotherms) at the nest [42,43]. Therefore, from 2019 to 2021, we deactivated motion sensors and used only time lapse settings (2019—1 min [60 photographs per h]; 2020–2021—5 min [12 photographs per h]) to photograph faunal associates at nests. We replaced camera memory cards (16 and 32 GB) every 3 (2016–2018), 6 (2019), and 21 (2020–2021) days, and photographs were downloaded onto an external hard drive. Camera batteries (Energizer Ultimate Lithium AA12, St. Louis, MO, USA) were replaced approximately every 6 (2016–2018), 18 (2019), and 28 (2020–2021) days. We placed cameras at nests within approximately 12 h to 4 days of oviposition, and they remained in place for different lengths of time depending on the year. In 2016–2018, each camera was removed after eggs in its corresponding nest hatched or were lost to predators. In 2019, all cameras remained armed at each nest (even if some nests had been predated) until the last successful clutch monitored that year had hatched (early September). Cameras deployed in June 2020 remained armed at each nest through the end of May 2021 (~one year). In June 2021, a single camera was placed at one nest and remained armed through late August. For the purposes of this study, only photographs taken during the months encompassing the alligator egg incubation period in South Carolina (June–September; [7]) were analyzed for nest associates. 

We examined each photograph for the presence of fauna on the nest or in the clearing surrounding the alligator nest mound. Animals observed in photographs were identified to the lowest possible taxonomic level. We classified photo records of nest-associated fauna as independent detections when two successive photographs indicated the arrival of a new species or individual animal at the nest or (when only a single species was observed in a string of successive images) on occasions when the same species was photographed after at least 1 h since the prior event [44,45]. We also recorded the primary behaviors/activities displayed by each individual nest associate photographed and assigned them to one of the following categories: (1) feeding/foraging—the animal probed or dug into the nest substrate, pursued prey on or near the nest, or actively consumed food items; (2) basking (reptiles only)—the animal laid or positioned itself on or adjacent to the nest for several minutes with little movement; (3) nesting—the animal deposited or attempted to deposit eggs on, in, or adjacent to the nest mound; (4) burrowing/shelter—the animal buried/concealed itself in nest material or constructed tunnels into or beneath the nest mound; (5) loafing—the animal slept, sat, stood, rested, defecated, urinated, and/or preened on or adjacent to the nest, apart from feeding and basking; and (6) traveling—the animal continually moved across the nest or nest site (used the nest site as a travel corridor). In addition to animals observed in game camera photographs, we also included any additional species encountered in person by researchers when visiting nest sites during the study period (June–September). Attending female and hatchling alligators were not included as nest associates in this study. Finally, although the presence of attending female alligators at nest sites likely reduces the occurrence of faunal associates during these periods, the overall impact is probably low, as, on average, female alligators at our study site only spend about 1% of the egg incubation period at the nest (Rainwater, Singh, Wilkinson, unpubl. data). 

## 3. Results

**Figure 1:**
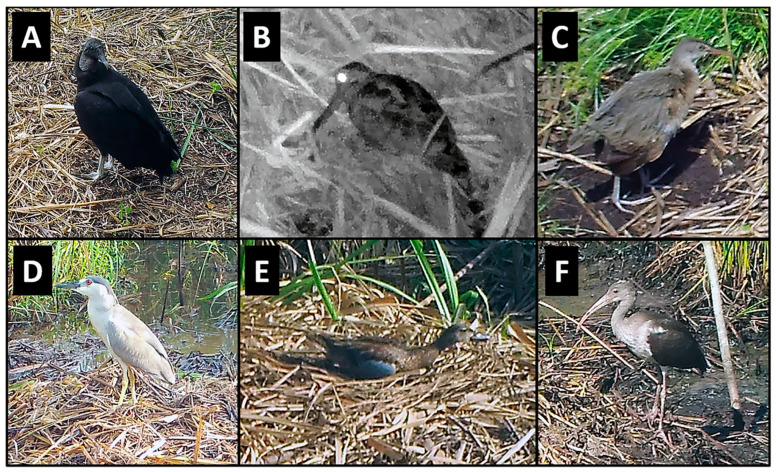
Examples of birds photographed at American alligator (*Alligator mississippiensis*) nests in coastal South Carolina, 2016–2021. (**A**) Black vulture (*Coragyps atratus*); (**B**) American woodcock (*Scolopax minor*); (**C**) clapper rail (*Rallus crepitans*); (**D**) black-crowned night heron (*Nycticorax nycticorax*); (**E**) blue-winged teal (*Anas discors*); (**F**) white ibis (*Eudocimus albus*).

**Figure 2:**
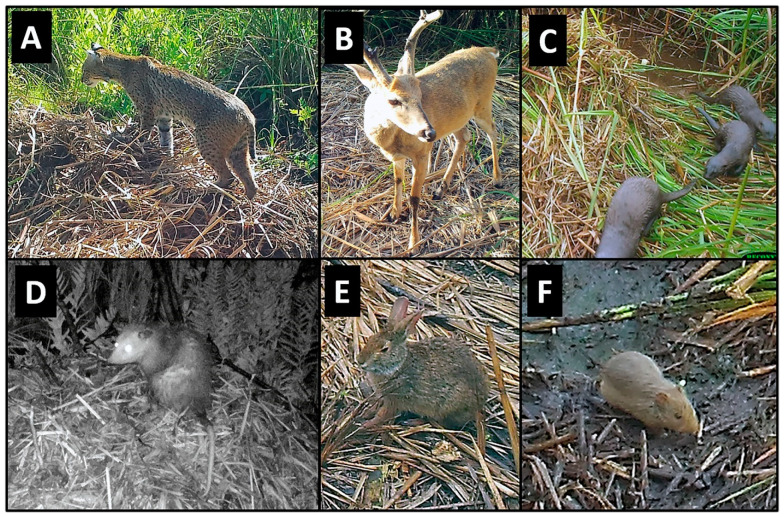
Examples of mammals photographed at American alligator (*Alligator mississippiensis*) nests in coastal South Carolina, 2016–2021. (**A**) Bobcat (*Lynx rufus*); (**B**) white-tailed deer (*Odocoileus virginianus*); (**C**) river otter (*Lontra canadensis*); (**D**) Virginia opossum (*Didelphis virginiana*); (**E**) marsh rabbit (*Sylvilagus palustris*); (**F**) marsh rice rat (*Oryzomys palustris*).

**Figure 3:**
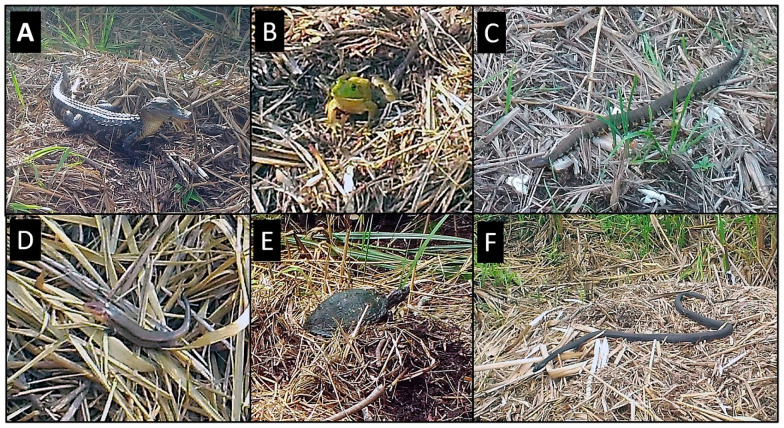
Examples of reptiles and amphibians photographed at American alligator (*Alligator mississippiensis*) nests in coastal South Carolina, 2016–2021. (**A**) American alligator; (**B**) American bullfrog/pig frog (*Lithobates catesbeianus*/*Lithobates grylio*); (**C**) eastern cottonmouth (*Agkistrodon piscivorus*); (**D**) broad-headed skink (*Plestiodon laticeps*); (**E**) yellow-bellied slider (*Trachemys scripta*); (**F**) eastern black racer (*Coluber constrictor*).

**Figure 4:**
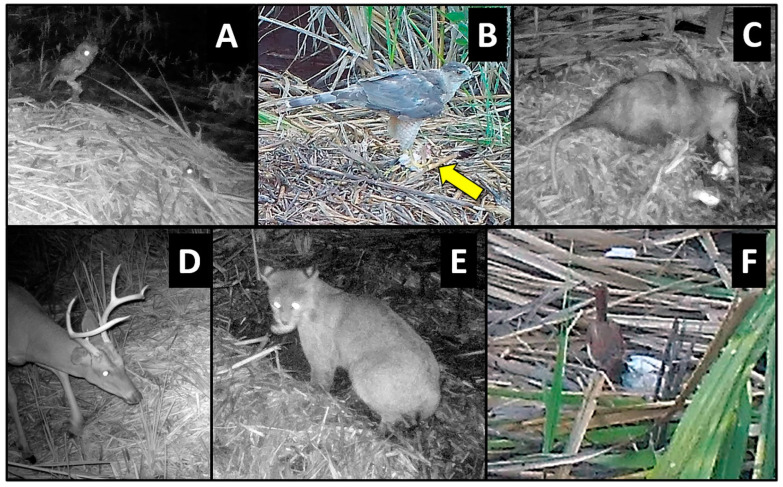
Examples of faunal associates feeding/foraging at American alligator (*Alligator mississippiensis*) nests in coastal South Carolina, 2016–2021. (**A**) Eastern screech owl (*Megascops asio*) watching an unidentified rodent (eyeshine at bottom right) on nest. (**B**) Cooper’s hawk (*Accipiter cooperii*) feeding on an unidentified bird (arrow). (**C**) Virginia opossum (*Didelphis virginiana*) scavenging alligator eggs following nest predation by raccoons (*Procyon lotor*); (**D**) white-tailed deer (*Odocoileus virginianus*) browsing on vegetation emerging from nest; (**E**) bobcat (*Lynx rufus*) with a hatching alligator egg in its mouth; (**F**) Carolina wren (*Thryothorus ludovicianus*) feeding on the remaining contents of a recently predated alligator egg.

We examined ~2.8 million game camera photographs (4545 trap days) for faunal associates at alligator nests during this study. A total of 78 alligator nests was monitored: 6 in 2016, 16 in 2017, 12 in 2018, 25 in 2019, 18 in 2020, and 1 in 2021. We documented a total of 81 species (79 vertebrates and 2 invertebrates) representing six taxonomic groups, including 48 birds (59.2%), 9 mammals (11.1%), 19 reptiles (23.4%), 3 amphibians (3.7%), 1 malacostracan (1.2%), and 1 insect (1.2%) (Table 1; Figure 1, Figure 2, Figure 3 and Figure 4). Not including individuals that could not be identified (*n* = 2069), a total of 1426 birds, 1494 mammals, 728 reptiles, 314 amphibians, and 7 invertebrates were photographed at alligator nest sites (Table 1). These animals (total identifiable associates = 3969) were independently detected using alligator nests and/or nest sites for a variety of life activities, including feeding/foraging (51.8%), basking (7.3%), traveling (29.3%), loafing (19.9%), burrowing (<1%), breeding (<1%), and possibly nesting (the sum of these percentages exceeds 100% because some individuals displayed more than one behavior). 

### 3.1. Birds

Of the 48 species of birds observed in photographs, the majority were passerines (45.8%) and wading birds (33.3%), but raptors (8.3%), non-passerine land birds (8.3%), and waterfowl (4.1%) were also present (Table 1; Figure 1). Most species were observed feeding/foraging (87.5%) and loafing (77.0%) on the nest mound or adjacent nest site, while a smaller proportion of birds (29.1%) was observed traveling (Table 1, Table 2). Passerines used nests and nest sites primarily for loafing, followed by feeding/foraging and traveling, whereas wading birds, raptors, and non-passerine land birds mostly used nests for feeding/foraging, followed by loafing and traveling (wading birds only) (Table 2). Waterfowl did not feed/forage at nests, but both species loafed, and one species traveled (Table 2). 

**Figure 5:**
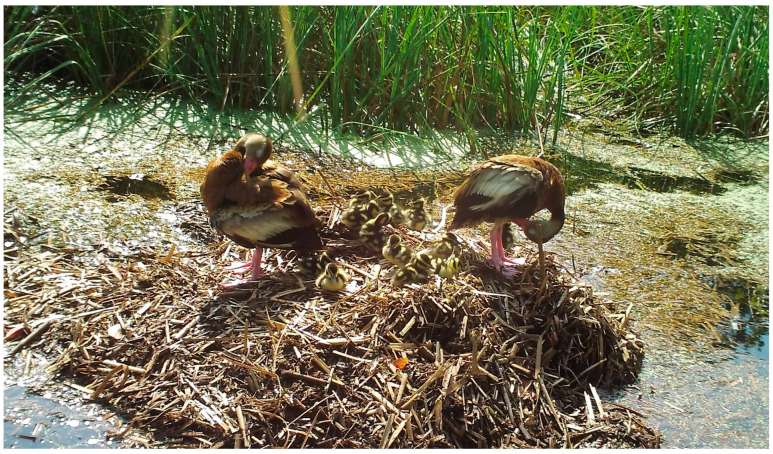
A breeding pair of black-bellied whistling ducks (*Dendrocygna autumalis*) and its brood standing on top of a flooded American alligator (*Alligator mississippiensis*) nest in coastal South Carolina, 2017. Both adult ducks are preening.

**Figure 6:**
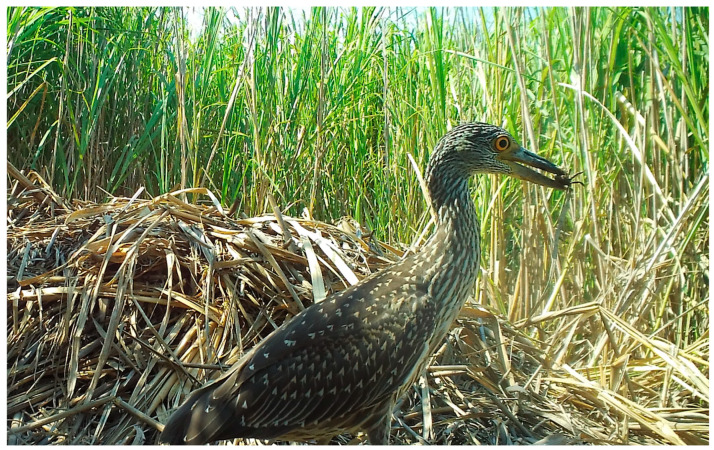
An immature yellow-crowned night heron (*Nyctanassa violacea*) feeding on a fiddler crab (*Minuca* or *Leptuca* sp.) at an American alligator (*Alligator mississippiensis*) nest site (note nest mound in background) in coastal South Carolina, 2017.

**Figure 7:**
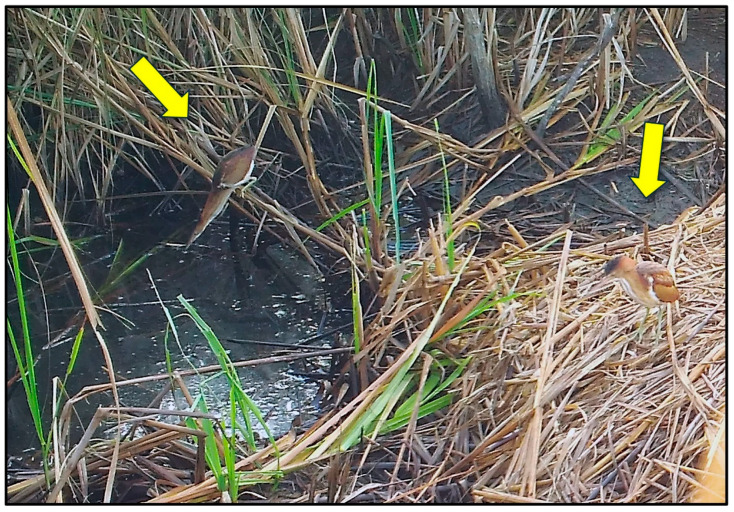
Two least bitterns (*Ixobrychus exilis*) (arrows) foraging on an American alligator (*Alligator mississippiensis*) nest mound (right) and in the associated guard hole (left) in coastal South Carolina, 2019.

Loafing was primarily characterized by standing (but not probing or feeding) on the nest mound for several minutes at a time (Figure 1); however, in some cases, birds sat/rested on their ventrum (e.g., blue-winged teal [*Anas discors*], Figure 1) or preened (e.g., black-bellied whistling ducks [*Dendrocygna autumnalis*], Figure 5) for extended periods while on the nest mound. Feeding/foraging consisted primarily of birds probing with their bills in and around nests or perching on protruding or overhanging vegetation, presumably searching for small vertebrates and invertebrates. A single downy woodpecker (*Picoides pubescens*) and northern flicker (*Colaptes auratus*) both briefly perched on stumps or limbs protruding from nest mounds, appearing to search for food in the decaying wood and nest material, respectively. Eastern screech owls (*Megascops asio*) commonly visited alligator nests at night, and one series of photographs showed an owl a few centimeters from the position occupied by an unidentified frog in the prior photograph (1 min before), suggesting pursuit and probable consumption of the frog. In a similar series of images, a screech owl was perched on vegetation at the edge of a nest and appeared to be watching an unidentified rodent less than 1 m away on top of the nest (Figure 4A). In the following photograph (≤1 min later), both animals were absent, suggesting the owl may have pursued the rodent. Another series of photographs showed a Cooper’s hawk (*Accipiter cooperii*) using an alligator nest mound as a feeding platform for the consumption of an unidentified bird (Figure 4B). In another set of consecutive images, a Carolina wren (*Thryothorus ludovicianus*) left its perch next to the nest, flew to the ground, and inserted its head into the open end of a recently predated (by raccoons) alligator egg, presumably feeding on remaining egg contents (Figure 4F). Another set of pictures documented an immature yellow-crowned night heron (*Nyctanassa violacea*) feeding on several fiddler crabs (*Minuca* or *Leptuca* spp.) at the nest site (Figure 6). And, one series of photographs showed two least bitterns (*Ixobrychus exilis*) foraging on an alligator nest mound and in the adjacent guard hole (Figure 7).

### 3.2. Mammals

**Figure 8:**
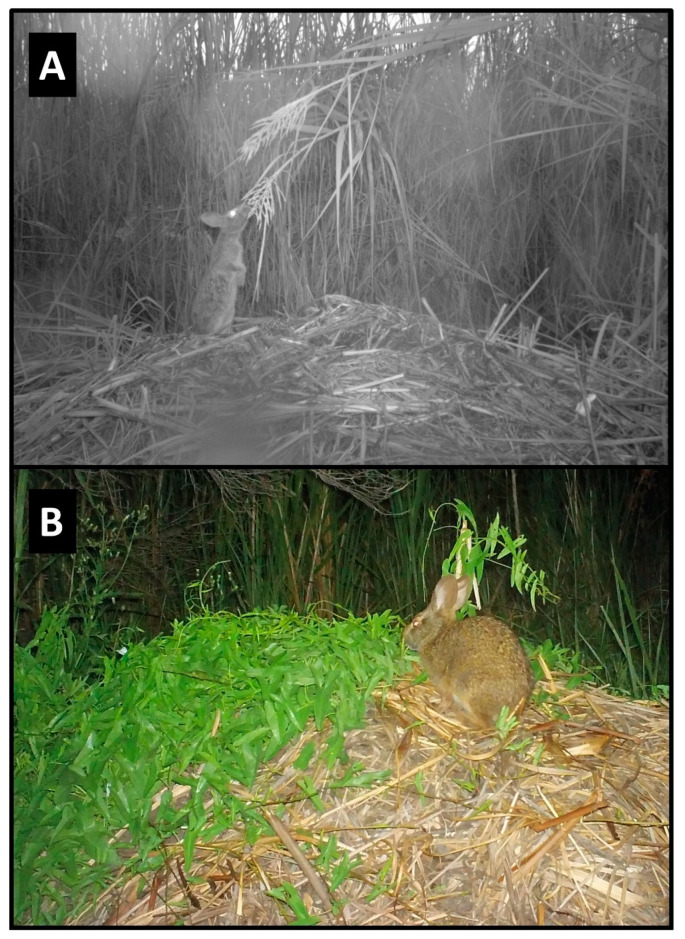
Marsh rabbits (*Sylvilagus palustris)* feeding on vegetation at American alligator (*Alligator mississippiensis*) nests in coastal South Carolina. (**A**) A rabbit uses a nest mound to access and browse overhanging tall cordgrass (*Spartina cynosuroides*) in 2017. (**B**) A rabbit feeds on newly sprouted vegetation growing atop a nest mound in 2016.

**Figure 9:**
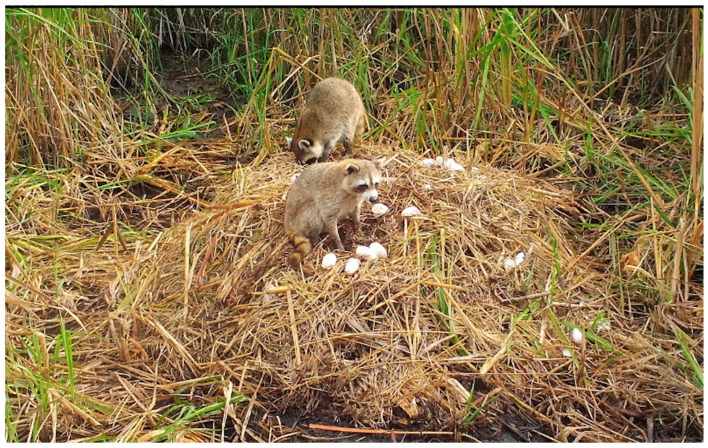
Two raccoons (*Procyon lotor*) predating an American alligator (*Alligator mississippiensis*) nest in coastal South Carolina, 2019.

**Figure 10:**
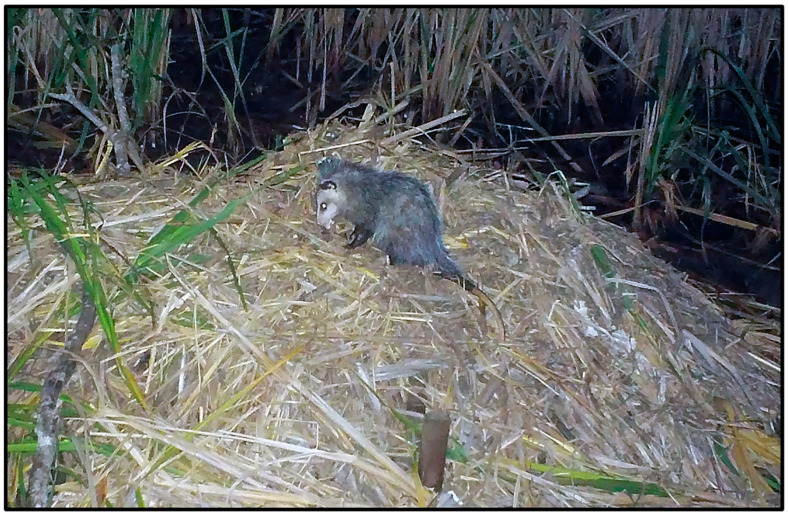
A Virginia opossum (*Didelphis virginiana*) feeding on an egg it removed from an American alligator (*Alligator mississippiensis*) nest in coastal South Carolina, 2019. This was one of only two instances during the study in which an opossum opened the nest, removed an egg, and consumed it. In both cases, only one egg was removed, and the remaining clutch later hatched successfully.

Nine mammal species were photographed at alligator nests (Table 1; Figure 2). Most were observed traveling (88.8%) and feeding/foraging (77.7%) on and adjacent to nests, while fewer were observed loafing (44.4%) and burrowing into nest material (11.1%) (Table 1, Table 2). Most mammals exhibited more than one of these behaviors, except for a single wood rat (*Neotoma floridana*) and four river otters (*Lontra canadensis*), which were observed feeding/foraging and traveling only, respectively (Table 2). Marsh rabbits (*Sylvilagus palustris*) and marsh rice rats (*Oryzomys palustris*) were commonly observed on and adjacent to alligator nests (Figure 2E,F), while white-tailed deer (*Odocoileus virginianus*) and other unidentified rats (likely *Rattus* sp. but possibly *Sigmodon hispidus*, which are both morphologically similar to *Oryzomys* and may occasionally be found in coastal wetlands [46,47,48]) were also seen but less frequently. All of these species were photographed feeding on vegetation on or adjacent to nests, particularly new vines and other plants emerging from nest mounds late in the incubation period (Figure 4D and Figure 8). Rice rats were also occasionally observed scavenging on egg remains following nest predation events. Raccoons were the primary predators of alligator nests (Figure 9) and were responsible for 42.8% (33 of 77 nests; the remaining clutch was collected by researchers as part of a separate study) of nest predation observed during this investigation. In addition, Virginia opossums opened nests on two occasions and consumed one egg from each nest (Figure 10); in both cases, the opossums did not return, and the remaining eggs later hatched successfully. More commonly, however, opossums scavenged eggs after nests had been opened by raccoons (Figure 4C). 

**Figure 11:**
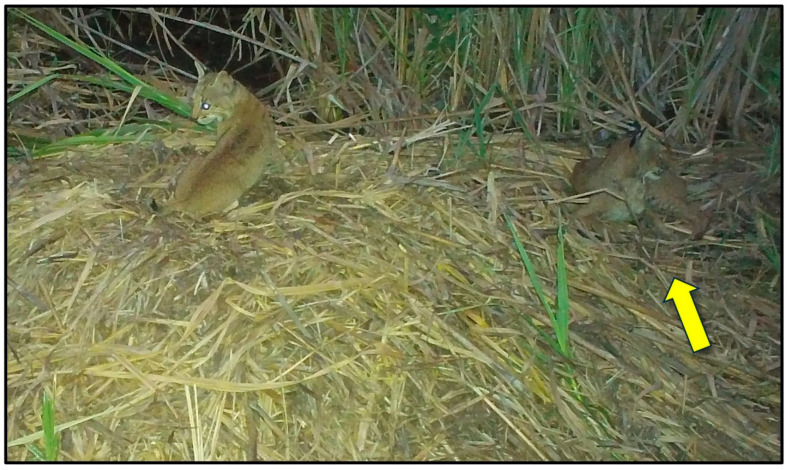
An adult female bobcat (*Lynx rufus*) (arrow) lying adjacent to an American alligator (*Alligator mississippiensis*) nest while her cub sits on top of the nest mound in coastal South Carolina, 2019.

On two occasions, we observed events suggesting bobcats (*Lynx rufus*) were consuming hatchling alligators as they emerged from nests. In both cases, bobcats appeared at night after the attending female alligator had opened the nest and was transporting hatchlings to water. Bobcats carefully investigated the nests from a short distance (≤1 m) but disappeared from the camera frame when the female alligator returned. In one series of photographs, a bobcat approached within ~0.5 m of a recently hatched neonate lying atop the nest and appeared to be looking directly at it. In the following photograph (≤1 min later), both animals were gone, and the nest material between the two had been disturbed, suggesting (but not confirming) that the bobcat had moved forward and taken the hatchling. Most tellingly, both bobcats were photographed grasping alligator eggs in their mouths (Figure 4E). Each egg was removed by bobcats during hatching from the recently opened (by the attending female alligator) egg chamber and likely contained an emerging or yet-to-emerge hatchling. Bobcats were never observed excavating unopened nests. In addition to feeding/foraging, adult bobcats also used alligator nest sites for loafing, occasionally being photographed standing or sitting on or adjacent to nest mounds. In 2019, an adult female bobcat lay next to an alligator nest for ~25 min while her cub crawled and walked on the nest mound (Figure 11). 

River otters were occasionally observed passing by nests (Figure 2C) but overall spent little time at nest sites. One eastern gray squirrel (*Sciurus carolinensis*) appeared to be drinking water adjacent to a nest. On one occasion, we observed a marsh rice rat burrowing into dead vegetation (excess nest material, *S*. *cynosuroides*) directly adjacent to an alligator nest, but we were unable to determine if this behavior was related to feeding/foraging, access to shelter, or both. Following hatching and the departure of hatchlings from the nest, we observed raccoons, opossums, and rice rats feeding on unhatched (infertile or undeveloped) eggs or contents adhering to the shells of hatched eggs.

### 3.3. Reptiles

Nineteen reptile species were observed at alligator nests, including 1 crocodilian (5.2%), 2 turtle (10.5%), twelve snake (63.1%), and 4 lizard (21.0%) species (Table 1; Figure 3). Most reptiles were observed traveling (84.2%) across the nest and nest site, followed by basking (42.1%), feeding/foraging (26.3%), and burrowing into nest material (26.3%). Alligators (excluding attending females and hatchlings) primarily traveled and basked on or adjacent to nests, and a small number appeared to pursue or feed on prey (Table 2), which included a snake or glass lizard (*Ophisaurus ventralis*) and several frogs. Over the course of the study, 14 alligators (excluding attending females and hatchlings) were visible in guard holes associated with a nest, and one animal defecated at the nest site. All snake species were observed traveling on or by nests, while basking and feeding/foraging were observed for only three species each (Table 2). A series of consecutive photographs showed a banded watersnake (*Nerodia fasciata*) on a nest pursuing an unidentified lizard, as well as a juvenile eastern rat snake (*Pantherophis alleghaniensis*) and a ribbon snake (*Thamnophis sauritus*) pursuing unidentified frogs/toads. Three snake species burrowed into alligator nests (Table 2). All lizard species were observed basking on alligator nests, and three species traveled across the nest/nest site (Table 2). Glass lizards also foraged/fed at nests and burrowed into nest material (Table 2). Both turtle species traveled at alligator nest sites, and one species each basked on (yellow-bellied slider [*Trachemys scripta*]) and burrowed into (eastern mud turtle [*Kinosternon subrubrum*]) nests (Table 2). The mud turtle partially burrowed into the side of a nest (the posterior of the turtle was concealed in nest material) and remained in place for ~87 min. The slider also scraped and dug into a different alligator nest (Figure 3E) but did not burrow. Rather, the turtle crawled to the top of the nest, positioned its posterior into nest material, and remained in place for ~5 min before departing.

### 3.4. Amphibians

**Figure 12:**
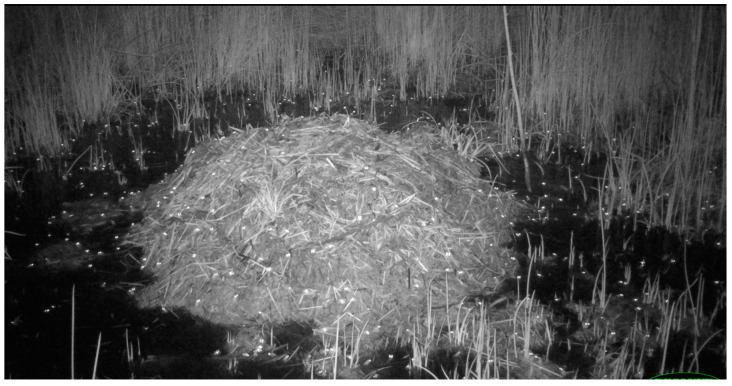
A large congregation of frogs (note eyeshine), possibly multiple species, on and around the nest of an American alligator (*Alligator mississippiensis*) in coastal South Carolina, 2019.

Three species of amphibians, all frogs, were identified on alligator nests (Table 1), and all three species were observed feeding/foraging and traveling (Table 2). Of all frogs photographed, 75.7% were feeding/foraging, 30.5% were traveling, and 4.7% were burrowing/sheltering. In general, frogs were abundant on and around nests, particularly at night, but in most cases were too small to identify to species in photographs. In addition, because American bullfrogs (*Lithobates catesbeiana*) and pig frogs (*L. grylio*) are morphologically similar and sympatric in the study area [49], we were unable to distinguish the two species in photographs; therefore, for the purposes of this study, we combined these species into a single group (bullfrog/pig frog) (Table 1, Table 2). On some occasions, large numbers of unidentified frogs, possibly representing multiple species, were present on alligator nests and in the surrounding water (Figure 12). In these cases, we were unable to determine if frogs were foraging, breeding, or both. On separate occasions (and at different nests), southern leopard frogs (*L. sphenocephala*) were photographed vocalizing (inflated vocal sacs) on a nest, in amplexus on top of a nest, and crawling into the side of a nest mound and sheltering there most of the day. 

### 3.5. Invertebrates

**Figure 13:**
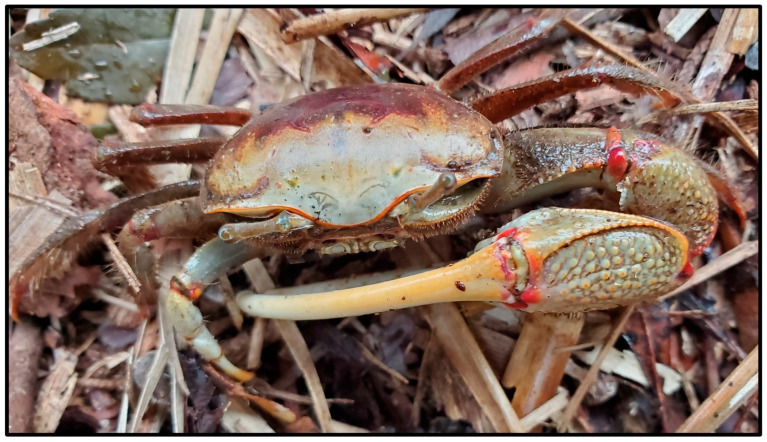
A red-jointed fiddler crab (*Minuca minax*) at the edge of an American alligator (*Alligator mississippiensis*) nest in coastal South Carolina, 2021.

**Figure 14:**
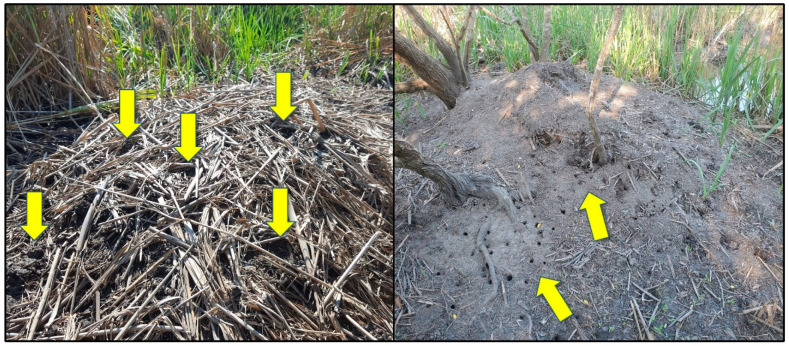
Openings to fiddler crab burrows (arrows) on active (**left**) and inactive (**right**) American alligator (*Alligator mississippiensis*) nests in coastal South Carolina, 2017 and 2021, respectively.

Multiple invertebrate species were observed associated with alligator nests in this study; however, most were insects and could not be confidently identified from game camera photographs (e.g., dragonflies [Odonata] and butterflies [Lepidoptera]). In addition, there were undoubtedly numerous insects on and within nest material that were not visible in photographs, and because we searched only the egg chamber of each alligator nest, faunal associates elsewhere in the mound potentially went undetected. The two most conspicuous identifiable invertebrates observed at alligator nests were fire ants and fiddler crabs (*Minuca* or *Leptuca* spp.) (Table 1). Neither were observed in game camera photographs (apart from crabs being consumed by birds) but were rather encountered by researchers when visiting nests. We only kept records regarding the presence of fire ants at nests during 2019, and these insects were found in 2 of the 25 (8%) nests monitored that year. Fire ants were observed nesting both inside and on top of the alligator nest mound. Fiddler crabs were observed only at nests adjacent to brackish water sources and were seen moving around the nest site and excavating burrows beneath and into nest mounds (Figure 13 and Figure 14).

## 4. Discussion

In this study, we aimed to identify fauna associated with American Alligator nests and examine how different species use nest mounds and nest sites. Our results indicate that a diverse assemblage of vertebrates (and some invertebrates) use alligator nest sites for a variety of life activities during the egg incubation period (June–September). This study is the first systematic assessment of fauna associated with American Alligator nests near the species’ northern distributional limit and only the third such investigation for alligators range-wide [10,18]. In addition, while previous studies have provided lists of species present at crocodilian nests and their species-specific activities/behaviors, our study is the first to quantify the number of individuals within a species present at nests and the activity/behavior of each individual. Most accounts of fauna associated with crocodilian nests are the result of chance encounters and observations; however, the use of automated game cameras (as performed in this study) allows for much longer and continuous monitoring, with minimal disturbance to nest sites compared to that incurred by the presence of human observers, thereby greatly increasing the detection probability of faunal associates. 

All 48 bird species recorded in this study used alligator nests and nest sites for either feeding/foraging or loafing, and most birds engaged in both activities. In one of the few other studies using game cameras to examine fauna associated with alligator nests, Merchant et al. [10] reported 22 bird species, primarily wading birds, at nests in southwest Louisiana and southeast Texas. Similar to our findings, these species all fed directly on invertebrates associated with nests or used nest mounds as staging grounds to feed in nearby water [10]. Further, González-Desales et al. [11] reported feeding by 8 of 11 (72.7%) avian species photographed at nest mounds of Morelet’s crocodiles (*Crocodylus moreletii*) in Mexico. Although several species of birds (primarily crows, ravens, and vultures; [50,51]) are known to feed on crocodilian eggs, and egg consumption by some wrens has been documented [52], our observation of a Carolina wren feeding on the contents of a recently predated alligator egg is, to the best of our knowledge, the first report of egg consumption by this species and the first record of crocodilian egg consumption by a passerine. Despite loafing being one of the primary activities exhibited by birds at alligator nests in our study, this behavior has not been reported for birds in previous studies of crocodilian nest associates [10,11,12]. However, this may be due to differences in the way feeding/foraging behavior was defined among the studies; in our study, activities such as sitting and standing (when not associated with probing, digging, and pursuing/consuming prey) were categorized as loafing, while in other studies, these activities may have been considered components of feeding/foraging behavior. In addition, Platt et al. [12] found multiple black-bellied whistling duck nests on the ground close (<1 to 5 m) to Morelet’s crocodile nests in Belize and speculated that nesting birds may benefit from the protection afforded by attending female crocodilians. We did not observe birds nesting near alligator nests in the present study, but it is possible bird nests were present in some cases, obscured by vegetation or out of the camera field of view.

Most mammals observed in this study also used alligator nests for feeding/foraging, which often involved predation of alligator eggs from the nest mound. Raccoons were the primary predator of alligator nests at our study site, similar to previous reports from coastal South Carolina [7]. Indeed, raccoon predation is the foremost cause of nest failure throughout the range of the American alligator [10,13,15,17,19,22,32,34,53,54,55,56]. Our observations of Virginia opossums and bobcats feeding at alligator nests are particularly noteworthy. McIlhenny [15] (p. 89) noted that “nests of alligators are robbed for their eggs” by opossums, but it is unclear if he observed opossums excavating clutches or scavenging eggs after nests were opened by other predators. González-Desales et al. [11] recently reported predation of Morelet’s crocodile eggs by a Virginia opossum, but in this case, the opossum took eggs from the nest after it had been previously opened by a raccoon. To the best of our knowledge, our study is the first report of Virginia opossums opening intact alligator nests and removing eggs for consumption. The only other similar report was by Torralvo et al. [57], who observed a single common opossum (*D*. *marsupialis*) opening and taking eggs from a black caiman (*Melanosuchus niger*) nest in Brazil. Photo records from our study suggest that initial predation (i.e., the first opening of a nest followed by egg consumption) of alligator nests by opossums is infrequent and often results in the loss of only a small portion of the clutch. Finally, although felids are commonly known to prey on crocodilians [50] and bobcats are suspected predators of alligator hatchlings [10], our study is the first confirmed report of the latter. 

Most reptiles photographed in this study were observed traveling across the nest site and basking on nests, although some individuals (i.e., banded watersnakes and ribbon snakes) were seen feeding/foraging (pursuing prey). Basking was also a predominant behavior exhibited by reptiles on alligator nests in Louisiana and Texas [10] and Morelet’s crocodile nests in Mexico [11]. In addition, all reptiles observed in the former study [10] also used alligator nests as egg deposition sites. Many reptiles have been reported to deposit eggs within crocodilian nests (reviewed by Escobedo-Galván et al. [58]), including lizards [11,12,25,26,27,28,59,60], snakes [10,12,26,29], and turtles [10,12,25,26,30,31,33,61]. Crocodilian nest mounds are favorable nesting sites for other reptiles because they provide adequate nesting material, are elevated (reducing the risk of egg inundation from flooding) [27,33], exhibit relatively high and stable internal temperatures and humidity (favorable incubation conditions for reptile eggs [25,29,62,63]), and are defended against predators by female crocodilians, which may provide incidental protection to clutches of other reptiles [12,16]. In this study, we did not search for or incidentally encounter eggs of other reptiles in alligator nests. However, our observation of a mud turtle partially burrowed into the side of an alligator nest for almost 87 min suggests this animal may have deposited a clutch of eggs. In addition, the yellow-bellied slider we observed scraping and digging into an alligator nest suggests that these turtles may use alligator nest mounds as nesting sites in our study area. Indeed, both mud turtles and sliders have been documented nesting in alligator nests in other areas [10,31], so the occurrence of this phenomenon in coastal South Carolina is to be expected. Collectively, our observations of reptiles at alligator nests are consistent with those of González-Desales et al. [11], who reported traveling and basking as the primary activities of reptiles at crocodilian nests in Mexico.

Frogs were common at alligator nests during this study, although most could not be identified to species (Table 1). We speculate frogs in coastal habitats use alligator nests and surrounding nest sites primarily for feeding/foraging, breeding, and traveling. In contrast to our study, frogs have rarely been reported as crocodilian nest associates in other investigations. González-Desales et al. [11] reported Mesoamerican cane toads (*Rhinella horribilis*) traveling and basking at nests of American (*Crocodylus acutus*) and Morelet’s crocodile nests in Mexico. In the only other study to use game cameras to examine fauna associated with alligator nests, Merchant et al. [10] reported no amphibians at nests in southwestern Louisiana and southeastern Texas. The only other amphibians reported to use alligator nests are two-toed amphiumas, who have been found to deposit their egg clutches in alligator nest mounds in Florida [23,24].

Although ants commonly colonize nests of both mound- and hole-nesting crocodilians [12,19,22,39,64,65,66,67], we only found these insects in 8% of nests monitored (2019). Wilkinson [7] and Platt et al. [19] found similar but slightly higher fire ant colonization rates (17% and 11%) in alligator nests examined in coastal South Carolina and southeastern Louisiana, respectively, while Reagan et al. [39] observed a considerably greater ant colonization rate (36%) in nests in southwestern Louisiana. However, fire ant colonization of crocodilian nests may occur 10–14 days following egg-laying [12], and since, in this study, we never opened nests after confirming oviposition, it is possible we underestimated the presence of fire ants in alligator nest mounds in later stages of incubation. The ant colonization process in crocodilian nests and subsequent effects of ant colonies on hatchling crocodilian fitness and nest success are poorly understood [12]; however, previous studies suggest that the presence of ant colonies in crocodilian nests may represent a trade-off between the safety of the clutch (e.g., elimination of fungi, bacteria, and rotten eggs by ants in the nest; deterrence of predators) and ant-induced mortality of hatchlings (e.g., directly by killing hatchlings upon emergence from eggs; indirectly by discouraging nest attendance by maternal females, resulting in a failure to open the nest and release neonates) [12,22,39,67]. 

To the best of our knowledge, this study is the first to report fiddler crabs constructing burrows in alligator nests. Fiddler crabs are known to excavate two structurally and functionally distinct burrow types, which serve as refuges and breeding sites, respectively [68,69]. Since we were unable to identify all fiddler crab species observed at alligator nests or associate crab species with different burrow types, we can only speculate their use was for one or both purposes. Fiddler crab burrows are known to aerate marsh sediments and influence sediment oxygen dynamics [70]. This raises interesting questions regarding the potential effects of fiddler crab burrows constructed in alligator nests on internal gas flux and thermal dynamics and, subsequently, alligator egg incubation temperatures, hatchling sex ratios, and neonatal survival associated with those nests [71]. Overall, except for ants, virtually nothing is known regarding the invertebrate fauna of crocodilian nest mounds despite the potential of these large heaps of decomposing vegetation as an ideal, albeit ephemeral, habitat for a variety of species.

## 5. Conclusions

The results of this study indicate that numerous species of wildlife visit and use alligator nests for a variety of purposes in coastal South Carolina. In the only other study to employ game cameras to assess fauna associated with alligator nests, Merchant et al. [10] similarly recorded a diversity of birds, mammals, and reptiles associated with alligator nests in Louisiana and Texas. Differences in the number and composition of wildlife species detected between the two studies are at least partially due to differences in geographic location and alligator nesting habitat and, consequently, differences in species diversity and abundance among the study areas. In addition, the preponderance of birds, reptiles, and amphibians observed at alligator nests in South Carolina compared to Louisiana and Texas may also reflect the different game camera settings (trapping effort) used in the two studies. While Merchant et al. [10] used a 5 min time lapse for game cameras, we used a combination of motion sensors, 5 min time lapse, and 1 min time lapse, the latter of which generated ~2.3 million photographs in 2019 alone. Therefore, many more photographs were taken in our study, increasing the probability of detecting faunal associates, particularly individuals that were only present for relatively brief periods.

Whether interactions between alligator nests and faunal associates are largely facultative or obligatory is mostly unknown [2], and additional studies focusing on this topic are needed to adequately examine the role of alligators as potential keystone species. In addition, further investigation is needed to determine the net impacts of alligator nest-faunal associate interactions [2]. While it is assumed crocodilian nest mounds provide significant ecological services to associated fauna (e.g., feeding, basking, breeding, nesting, and sheltering sites), few supporting empirical data are available [2,12]. In fact, some studies indicate these associations can be detrimental to commensal fauna, as some species risk being killed and eaten by attending female crocodilians [25,33,59), and eggs deposited by commensals may be damaged or destroyed by female crocodilians during nest construction, maintenance, and post-hatching excavation [18,33]. Likewise, sharing nesting space with other animals may be disadvantageous for crocodilians due to disturbance to nesting females [59], damage to crocodilian eggs by commensal nest dwellers [18.33], and increased egg predation rates due to greater olfactory cues [32,37]. Until additional empirical studies are conducted, the true nature of the relationships between crocodilians and nest associates will remain largely speculative [12].

## Figures and Tables

**Table 1:** Fauna associated with American alligator (*Alligator mississippiensis*) nest mounds/sites in coastal South Carolina (2016–2021) and their primary behaviors. Percentages in parentheses indicate frequency of occurrence of each species at all nests monitored (*n* = 78).

Animal Group	Common Name	Scientific Name	Number Recorded	# Nests Where Recorded	Behavior
Vertebrates					
Birds					
Passerines					
	Blue-gray Gnatcatcher	*Polioptila caerulea*	5	4 (5%)	Feeding/Foraging, Loafing
	Blue Grosbeak	*Passerina caerulea*	3	3 (3%)	Loafing
	Blue Jay	*Cyanocitta cristata*	2	2 (2%)	Loafing
	Boat-tailed Grackle	*Quiscalus major*	6	4 (5%)	Feeding/Foraging, Loafing
	Brown-headed Cowbird	*Molothrus ater*	1	1 (1%)	Feeding/Foraging
	Brown Thrasher	*Toxostoma rufum*	10	7 (8%)	Feeding/Foraging, Loafing
	Carolina Wren	*Thryothorus ludovicianus*	48	18 (23%)	Feeding/Foraging, Traveling, Loafing
	Common Grackle	*Quiscalus quiscula*	18	5 (6%)	Feeding/Foraging, Loafing
	Common Yellowthroat	*Geothlypis trichas*	45	15 (19%)	Feeding/Foraging, Loafing
	Eastern Kingbird	*Tyrannus tyrannus*	67	2 (2%)	Feeding/Foraging, Loafing
	Eastern Towhee	*Pipilo erythrophthalmus*	4	3 (3%)	Feeding/Foraging, Loafing
	Gray Catbird	*Dumatella carolinensis*	3	1 (1%)	Feeding/Foraging
	Great Crested Flycatcher	*Myiarchus crinitus*	7	5 (6%)	Feeding/Foraging, Loafing
	Northern Cardinal	*Cardinalis cardinalis*	165	21 (26%)	Feeding/Foraging, Loafing, Traveling
	Northern Mockingbird	*Mimus polyglottos*	27	4 (5%)	Feeding/Foraging, Loafing
	Northern Waterthrush	*Parkesia noveboracensis*	10	6 (7%)	Feeding/Foraging, Loafing
	Orchard Oriole	*Icterus spurirus*	4	2 (2%)	Feeding/Foraging, Loafing
	Painted Bunting	*Passerina ciris*	58	15 (19%)	Feeding/Foraging, Loafing
	Palm Warbler	*Setophaga palmarum*	2	2 (2%)	Feeding/Foraging, Loafing
	Prairie Warbler	*Setophaga discolor*	1	1 (1%)	Loafing
	Red-winged Blackbird	*Agelaius phoeniceus*	318	36 (46%)	Feeding/Foraging, Traveling, Loafing
	White-eyed Vireo	*Vireo griseus*	1	1 (1%)	Loafing
	Unidentified Passerine ^1^	Unknown	169	34 (43%)	Feeding/Foraging, Traveling, Loafing
	Unidentified Waterthrush ^1^	*Parkesia sp.*	6	5 (6%)	Feeding/Foraging, Loafing
Wading birds					
	American Bittern	*Botaurus lentiginosus*	1	1 (1%)	Feeding/Foraging
	American Woodcock	*Scolopax minor*	10	3 (3%)	Feeding/Foraging
	Black-crowned Night Heron	*Nycticorax nycticorax*	24	5 (6%)	Feeding/Foraging, Loafing
	Clapper Rail	*Rallus crepitans*	125	16 (20%)	Feeding/Foraging, Traveling, Loafing
	Common Gallinule	*Gallinula galeata*	46	6 (7%)	Feeding/Foraging, Traveling, Loafing
	Great Blue Heron	*Ardea herodias*	4	2 (2%)	Feeding/Foraging, Traveling, Loafing
	Great Egret	*Ardea alba*	4	4 (5%)	Feeding/Foraging, Traveling, Loafing
	Green Heron	*Butorides virescens*	88	16 (20%)	Feeding/Foraging, Traveling, Loafing
	King Rail	*Rallus elegans*	11	3 (3%)	Feeding/Foraging, Loafing
	Least Bittern	*Ixobrychus exilis*	45	13 (16%)	Feeding/Foraging, Traveling, Loafing
	Little Blue Heron	*Egretta caerulea*	28	3 (3%)	Feeding/Foraging, Traveling, Loafing
	Snowy Egret	*Egretta thula*	7	5 (6%)	Feeding/Foraging, Traveling
	Sora	*Porzana carolina*	4	1 (1%)	Feeding/Foraging
	Tricolored Heron	*Egretta tricolor*	48	5 (6%)	Feeding/Foraging, Traveling, Loafing
	White Ibis	*Eudocimus albus*	40	5 (6%)	Feeding/Foraging, Loafing
	Yellow-crowned Night Heron	*Nyctanassa violacea*	13	3 (3%)	Feeding/Foraging, Traveling, Loafing
	Unidentified Wading Bird ^1^	Unknown	32	13 (16%)	Feeding/Foraging, Traveling, Loafing
Raptors					
	Black Vulture	*Coragyps atratus*	5	2 (2%)	Feeding/Foraging, Loafing
	Cooper’s Hawk	*Accipiter cooperii*	1	1 (1%)	Feeding/Foraging
	Eastern Screech Owl	*Megascops asio*	69	14 (17%)	Feeding/Foraging, Loafing
	Red-shouldered Hawk	*Buteo lineatus*	1	1 (1%)	Feeding/Foraging
Non-passerine land birds					
	Common Ground Dove	*Columbina passerina*	2	1 (1%)	Feeding/Foraging
	Downy Woodpecker	*Picoides pubescens*	1	1 (1%)	Feeding/Foraging
	Mourning Dove	*Zenaida macroura*	25	2 (2%)	Feeding/Foraging, Loafing
	Northern Flicker	*Colaptes auratus*	1	1 (1%)	Feeding/Foraging
Waterfowl					
	Black-bellied Whistling Duck	*Dendrocygna autumnalis*	14	1 (1%)	Loafing
	Blue-winged Teal	*Anas discors*	4	2 (2%)	Traveling, Loafing
Other unidentified birds ^1^					
	Unknown	Unknown	6	4 (5%)	Feeding/Foraging, Traveling, Loafing
Total birds			1639		
Mammals	Bobcat	*Lynx rufus*	17	7 (8%)	Feeding/Foraging, Traveling, Loafing
	Eastern Gray Squirrel	*Sciurus carolinensis*	1	1 (1%)	Traveling
	Eastern Woodrat	*Neotoma floridana *	1	1 (1%)	Feeding/Foraging
	Marsh Rabbit	*Sylvilagus palustris*	422	50 (64%)	Feeding/Foraging, Traveling, Loafing
	Marsh Rice Rat	*Oryzomys palustris*	183	31 (39%)	Feeding/Foraging, Burrowing/Shelter, Traveling
	Raccoon	*Procyon lotor*	648	50 (64%)	Feeding/Foraging, Traveling, Loafing
	River Otter	*Lontra canadensis*	4	2 (2%)	Traveling
	Virginia Opossum	*Didelphis virginiana*	152	24 (30%)	Feeding/Foraging, Traveling
	White-tailed Deer	*Odocoileus virginianus*	66	26 (33%)	Feeding/Foraging, Traveling, Loafing
	Unidentified Rat ^1^	Unknown	214	32 (41%)	Feeding/Foraging, Traveling
Total mammals			1708		
Reptiles					
Crocodilians					
	American Alligator ^2^	*Alligator mississippiensis*	229	29 (37%)	Feeding/Foraging, Basking, Traveling
Snakes					
	Banded Watersnake	*Nerodia fasciata*	25	16 (20%)	Feeding/Foraging, Basking, Traveling, Burrowing/Shelter
	Canebrake Rattlesnake	*Crotalus horridus*	1	1 (1%)	Traveling
	Copperhead	*Agkistrodon contortrix*	2	2 (2%)	Traveling
	Corn Snake	*Pantherophis guttatus*	3	3 (3%)	Traveling
	Eastern Black Racer	*Coluber constrictor*	188	37 (47%)	Basking, Burrowing/Shelter, Traveling
	Eastern Cottonmouth	*Agkistrodon piscivorus*	60	22 (28%)	Traveling
	Eastern Kingsnake	*Lampropeltis getula*	5	3 (3%)	Traveling
	Eastern Rat Snake	*Pantherophis alleghaniensis*	13	8 (10%)	Feeding/Foraging, Traveling
	Ribbon Snake	*Thamnophis sauritus*	65	24 (30%)	Feeding/Foraging, Basking, Burrowing/Shelter, Traveling
	Ringneck Snake	*Diadophis punctatus*	1	1 (1%)	Traveling
	Rough Green Snake	*Opheodrys aestivus*	3	3 (3%)	Traveling
	Scarlet Snake	*Cemophora coccinea*	2	1 (1%)	Traveling
	Unidentified Snake ^1^	Unknown	72	25 (32%)	Basking, Traveling, Burrowing/Shelter
Lizards					
	Broad-headed Skink	*Plestiodon laticeps*	38	15 (19%)	Basking, Traveling
	Eastern Glass Lizard	*Ophisaurus ventralis*	50	19 (24%)	Feeding/Foraging, Basking, Burrowing/Shelter, Traveling
	Green Anole	*Anolis carolinensis*	27	11 (14%)	Basking, Traveling
	Ground Skink	*Scincella lateralis*	1	1 (1%)	Basking
	Unidentified Skink ^1^	Unknown	108	26 (33%)	Traveling
	Unidentified Lizard ^1^	Unknown	21	11 (14%)	Basking, Traveling
Turtles					
	Eastern Mud Turtle	*Kinosternon subrubrum*	13	9 (11%)	Burrowing/Shelter (Nesting?), Traveling
	Yellow-bellied Slider	*Trachemys scripta*	2	2 (2%)	Basking, Traveling, Nesting?
Total reptiles			929		
Amphibians	American Bullfrog/Pig Frog	*Lithobates catesbeiana/Lithobates grylio*	29	9 (11%)	Feeding/Foraging, Traveling, Breeding?
	Green Tree Frog	*Hyla cinerea*	31	7 (8%)	Feeding/Foraging, Traveling, Breeding?
	Southern Leopard Frog	*Lithobates sphenocephala*	254	32 (41%)	Feeding/Foraging, Burrowing/Shelter, Traveling, Breeding
	Unidentified Frog ^1^	Unknown	1194	55 (70%)	Feeding/Foraging, Burrowing/Shelter, Traveling, Breeding?
Total amphibians			1508		
Invertebrates					
Malacostracans	Red-jointed Fiddler Crab	*Minuca minax*	7	1 [1%]	Traveling, Burrowing/Shelter
	Unidentified Fiddler Crab ^1^	*Minuca* or *Leptuca* spp.	247	17 [21%]	Traveling, Burrowing/Shelter
Insects	Fire Ant	*Solenopsis invicta*	Not quantified	Not quantified	Nesting
Total Invertebrates			254		

^1^ Not included when calculating total number of faunal associates observed (could possibly be a species already listed). ^2^ Attending female and hatchling alligators were not included as nest associates in this study.

**Table 2:** Number of individual American alligator (*Alligator mississippiensis*) nest associates that displayed specific behaviors during this study. Numbers in parentheses indicate the percentage of the total number of a given species that displayed the corresponding behavior. Because some individuals displayed more than one behavior, percentages may not sum to 100, and the total number of individuals per species may not agree with those in Table 1.

	Behavior					
Animal Group/ Common Name	Feeding/ Foraging	Basking	Nesting	Burrowing/ Shelter	Traveling	Loafing
Birds						
Passerines						
Blue-gray Gnatcatcher	2 (40%)					4 (80%)
Blue Grosbeak	–	–	–	–	–	3 (100%)
Blue Jay	–	–	–	–	–	2 (100%)
Boat-tailed Grackle	2 (33%)	–	–	–	–	4 (66%)
Brown-headed Cowbird	1 (100%)	–	–	–	–	–
Brown Thrasher	6 (60%)	–	–	–	–	5 (50%)
Carolina Wren	16 (33%)	–	–	–	1 (2%)	35 (72%)
Common Grackle	10 (55%)	–	–	–	–	8 (44%)
Common Yellowthroat	14 (31%)	–	–	–	–	36 (80%)
Eastern Kingbird	19 (28%)	–	–	–	–	63 (94%)
Eastern Towhee	1 (25%)	–	–	–	–	4 (100%)
Gray Catbird	3 (100%)	–	–	–	–	–
Great Crested Flycatcher	2 (28%)	–	–	–		6 (85%)
Northern Cardinal	40 (24%)	–	–	–	1 (0.6%)	137 (83%)
Northern Mockingbird	9 (33%)	–	–	–	–	19 (70%)
Northern Waterthrush	5 (50%)	–	–	–	–	5 (50%)
Orchard Oriole	2 (50%)	–	–	–	–	2 (50%)
Painted Bunting	10 (17%)	–	–	–	–	50 (86%)
Palm Warbler	1 (50%)	–	–	–	–	2 (100%)
Prairie Warbler	–	–	–	–	–	1 (100%)
Red-winged Blackbird	120 (37%)	–	–	–	5 (1%)	209 (65%)
White-eyed Vireo	–	–	–	–	–	1 (100%)
Wading birds						
American Bittern	1 (100%)	–	–	–	–	–
American Woodcock	10 (100%)	–	–	–	–	–
Black-crowned Night Heron	19 (79%)	–	–	–	–	10 (41%)
Clapper Rail	99 (79%)	–	–	–	36 (28%)	16 (12%)
Common Gallinule	33 (71%)	–	–	–	11 (23%)	3 (6%)
Great Blue Heron	2 (50%)	–	–	–	2 (50%)	2 (50%)
Great Egret	1 (25%)	–	–	–	2 (50%)	1 (25%)
Green Heron	78 (88%)	–	–	–	6 (6%)	29 (32%)
King Rail	11 (100%)	–	–	–	–	2 (18%)
Least Bittern	35 (77%)	–	–	–	7 (15%)	8 (17%)
Little Blue Heron	28 (100%)	–	–	–	1 (3%)	9 (32%)
Snowy Egret	6 (85%)	–	–	–	1 (14%)	–
Sora	4 (100%)	–	–	–	–	–
Tricolored Heron	23 (47%)	–	–	–	4 (8%)	26 (54%)
White Ibis	38 (95%)	–	–	–	–	11 (27%)
Yellow-crowned Night Heron	12 (92%)	–	–	–	1 (7%)	2 (15%)
Raptors						
Black Vulture	2 (40%)	–	–	–	–	5 (100%)
Cooper’s Hawk	1 (100%)	–	–	–	–	–
Eastern Screech Owl	60 (86%)	–	–	–	–	13 (18%)
Red-shouldered Hawk	1 (100%)	–	–	–	–	–
Non-passerine land birds						
Common Ground Dove	2 (100%)	–	–	–	–	–
Downy Woodpecker	1 (100%)	–	–	–	–	–
Mourning Dove	15 (60%)	–	–	–		14 (56%)
Northern Flicker	1 (100%)	–	–	–	–	–
Waterfowl						
Black-bellied Whistling Duck	–	–	–	–	–	14 (100%)
Blue-winged Teal	–	–	–	–	3 (75%)	1 (25%)
Mammals						
Bobcat	4 (23%)	–	–	–	7 (41%)	6 (35%)
Eastern Grey Squirrel	–	–	–	–	1 (100%)	–
Eastern Woodrat	1 (100%)	–	–	–	–	–
Marsh Rabbit	335 (79%)	–	–	–	120 (28%)	9 (2%)
Marsh Rice Rat	97 (53%)	–	–	4 (2%)	98 (53%)	–
Raccoon	522 (80%)	–	–	–	172 (26%)	9 (1%)
River Otter	–	–	–	–	4 (100%)	–
Virginia Opossum	67 (44%)				94 (61%)	
White-tailed Deer	29 (43%)	–	–	–	37 (56%)	6 (9%)
Reptiles						
Crocodilians						
American Alligator	6 (2%)	112 (48%)	–	–	123 (53%)	–
Snakes						
Banded Watersnake	4 (16%)	5 (20%)		1 (4%)	17 (68%)	–
Canebrake Rattlesnake	–	–	–	–	1 (100%)	–
Copperhead	–	–	–	–	2 (100%)	–
Corn Snake	–	–	–	–	3 (100%)	–
Eastern Black Racer	–	91 (48%)	–	1 (0.5%)	103 (54%)	–
Eastern Cottonmouth	–	–	–	–	60 (100%)	–
Eastern Kingsnake	–	–	–	–	5 (100%)	–
Eastern Rat Snake	2 (15%)	–	–	–	11 (84%)	–
Ribbon Snake	4 (6%)	20 (30%)	–	1 (1%)	45 (69%)	–
Ringneck Snake	–	–	–	–	1 (100%)	–
Rough Green Snake	–	–	–	–	3 (100%)	–
Scarlet Snake	–	–	–	–	2 (100%)	–
Lizards						
Broad-headed Skink	–	38 (71%)	–	–	12 (31%)	–
Eastern Glass Lizard	4 (8%)	2 (4%)	–	5 (10%)	44 (88%)	–
Green Anole	–	23 (85%)	–	–	5 (18%)	–
Ground Skink	–	1 (100%)	–	–	–	–
Turtles						
Eastern Mud Turtle	–	–	?	2 (15%)	11 (84%)	–
Yellow-bellied Slider	–	1 (50%)	?	–	1 (50%)	–
Amphibians						
American Bullfrog/Pig Frog	19 (65%)	–	–	–	11 (37%)	–
Green Tree Frog	23 (74%)	–	–	–	10 (32%)	–
Southern Leopard Frog	196 (77%)	–	–	15 (5%)	75 (29%)	–
Malacostracans						
Red-jointed Fiddler Crab	–	–	–	7 (100%)	7 (100%)	–

## Data Availability

The data presented in this study are available on request from the corresponding author. The data are not publicly available because they are associated with other projects that are ongoing or yet to be published.
